# “I go into crisis when …”: ethics of care and moral dilemmas in palliative care

**DOI:** 10.1186/s12904-019-0453-2

**Published:** 2019-08-09

**Authors:** Ludovica De Panfilis, Silvia Di Leo, Carlo Peruselli, Luca Ghirotto, Silvia Tanzi

**Affiliations:** 1 Unit of Bioethics, Azienda USL-IRCCS di Reggio Emilia, Via Amendola 2, 42122 Reggio Emilia, Italy; 2 Psycho-oncology Unit, Azienda USL-IRCCS di Reggio Emilia, Reggio Emilia, Italy; 3 Past President Italian Society of Palliative Care, Milano, Italy; 4 Azienda USL-IRCCS di Reggio Emilia, Reggio Emilia, Italy; 5 Palliative Care Unit, Azienda USL-IRCCS di Reggio Emilia, Reggio Emilia, Italy; 6 Clinical and Experimental Medicine PhD Program, University of Modena and Reggio Emilia, Modena, Italy

**Keywords:** Palliative care, Ethics of care, Communication, Cancer, Qualitative research

## Abstract

**Background:**

Recognising and knowing how to manage ethical issues and moral dilemmas can be considered an ethical skill. In this study, ethics of care is used as a theoretical framework and as a regulatory criterion in the relationship among healthcare professionals, patients with palliative care needs and family members.

This study is a part of a larger project aimed at developing and implementing a training programme on “ethical communication” addressed to professionals caring for patients with palliative care needs. The aim of this study was comprehending whether and how the ethics of care informs the way healthcare professionals make sense of and handle ethical issues in palliative care.

**Methods:**

Qualitative study employing a theoretically driven thematic analysis performed on semi-structured interviews.

The research was conducted in a clinical cancer centre in northern Italy. Eligible participants were physicians and nurses from eleven hospital wards who assisted patients with chronic advanced disease daily and had previously attended a 4-h training on palliative care held by the hospital Palliative Care Unit.

**Results:**

The researchers identified five themes: morality is providing global care; morality is knowing how to have a relationship with patients; morality is recognizing moral principles; moral dimension and communication; and moral dilemmas are individual conflicts.

**Conclusions:**

Ethics of care seems to emerge as a theoretical framework that includes the belief systems of healthcare professionals, especially those assisting patients with palliative care needs; moreover, it allows the values of both the patients and professionals to come to light through the relationship of care. Ethics of care is also appropriate as a framework for ethical training.

## Background

Palliative care is defined by the World Health Organization as “an approach that improves the quality of life of patients and their families facing problems associated with a life-threatening illness, through the prevention and relief of suffering by means of early identification and impeccable assessment and treatment of pain and other problems, physical, psychosocial and spiritual” [1]. Palliative care therefore requires many different competences, not only clinical but also relational, communicative and ethical [2].

Studies in the literature show that clear and honest communication about the diagnosis and prognosis of a fatal illness, which fully respects patients’ wishes and preferences, positively affects their quality of life and improves symptom management [3]. Good communication stems partly from innate quality and can improve with experience. Nevertheless, it can also be increased through specific training programmes that take into account all of the abovementioned domains. A number of studies have shown that healthcare professionals (HPs) recognise and address ethical issues and that their awareness of moral dilemmas that may arise in decision-making is part of effective communication [4, 5].

From the Greek word *ethos* meaning habit or custom, ethics is the branch of philosophy that concerns human behaviour, customs, and habits, particularly with reference to the rules of conduct and their justification [6].

Ethical debate in palliative care has focused on several and sometimes opposing approaches, among which is the classical deontological approach of principlism, “virtue” ethics, and ethics of care.

Principlism is based on principles originally proposed by Beauchamp and Childress [7]: autonomy (to give an individual the freedom to make his or her own choices), beneficence (to do good and to act with the best interests of the other person in mind), non-maleficence (to do no harm to people), and justice (to promote fairness and equality among individuals). Each principle relates to each of the other three principles; therefore, they should be ordered according to the criteria of priority for each individual case, with the ultimate aim of “the best interests of the patient” [7]. As this approach provides a valid basis for assessing the appropriateness of behaviours concerning morality, it may have some limitations concerning its full applicability in the medical context, above all within palliative care. Indeed, conveying the concept of the human being as a subject in its own right, fully aware, competent and independent, can be considered inadequate in medicine and health care, where human complexity and interpersonal relations need to be considered. Some authors argued that the four principles suggest that the approach is imperialist, inapplicable, inconsistent and inadequate [8]; others argued that the four-principle approach does not consider the role of emotional reactions as an integral part of our moral perceptions and decision making [9].

Virtue ethics may be identified as the ethical theory that emphasises virtues or moral character [10]. All forms of virtue ethics are based on two concepts, i.e., virtue and practical wisdom: virtue ethics is a framework that focuses on the moral character rather than the rightness of an action [9]; it provides a broader ethical analysis and encourages more flexible and creative solutions than principlism [11]. Its main limitations are putting too much emphasis on a person’s moral character and on cultural judgement of values, and the inability to provide decisional elements to support the choice [10].

The ethics of care theoretical framework [12] represents an interesting ethics approach for reading and analysing ethical issues and moral dilemmas in palliative care. In our view, it could represent not only a valid theoretical framework but also a guiding criterion in the relationship among HPs, patients with palliative care needs, and their families.

The central concept of this approach is care, conceived both as an action concretely expressed towards the other, and as a value that has the goal of being universally shared, beginning with the awareness of the fragility and vulnerability of the human condition [13]. Ethics of care recognises that human beings are interdependent, and for this reason, they need respect, protection, and care [14, 15]. Moreover, it highlights significant ethical aspects in the development of the relationship of care [14, 15]. From this perspective, every moral choice or ethical issue is conceived as inserted in a network of interpersonal relationships, nurtured by communication, since both illness and the patient experience can be considered as the products of a set of interconnections.

To deepen the theoretical relationship between ethics of care and palliative care, we reviewed the literature by combining the terms “care ethics” or “ethics of care” with “palliative care”. We retrieved articles [16–23] concerning two main topics, i.e., a) the need to set medical ethics on a new foundation by grounding it on a different set of values, such as compassion, heedfulness, vulnerability, and the integrity of the person; and b) the specificity of the moral dilemmas often arising in medical care and the need for approaching them with moral notions different from the classical moral theory [16–23].

Lachman discussed the use of the theory of care ethics to help nurses determine if they are applying this theory effectively in their practice [16]. After having described care ethics and its evolution through the main authors’ theories, he/she presents a case study to illustrate the philosophical approach of Joan Tronto [18]. Lachman assumes that a care orientation is fundamental to the nurse-patient relationship and that Joan Tronto’s version of the ethics of care must/might be implemented in the care relationship. Although this paper does not mention the Palliative care field, it provides the reader with a practical use of the ethics of care in a healthcare field.

William T. Branch has argued that ethics based on caring for the patient needs to be grounded on the patient/physician relationship, making it necessary to rely on the physician’s moral sensitivity [17]. He also argued that HPs can recognise patients’ wishes and preferences, but equally important is their capacity for compassion, as well as honesty, integrity and a sense of humility. He defines this approach as “the ethics of patient care” and assumes that building medical ethics on this foundation leads to a framework of caring ethics.

On these bases, Branch built a theoretical frame to include ethics of care as a suitable approach to palliative care.

In their research project “Practical ethics of Palliative care”, Hermsen and Ten Have [19] suggest that palliative care does not fit well into the classical biomedical model and that it can rather be considered a new philosophy of care, introducing new moral notions of wider relevance into the healthcare context. As a consequence, they argue that it is possible to identify a moral dimension that is specific to palliative care.

To widen the moral horizon and increase moral sensitivity, de Vries and Leget [20] introduce an ethical framework to address elderly patients with cancer. This ethical approach stems from the ethics of care because it focuses on the caring relationship. Authors compare ethics of care with principlism, which is the ethical theory predominant in contemporary medicine. As opposed to principlism, ethics of care underlines not only the attention to the patient context but also a broader comprehension of the illness and a different concept of autonomy [20].

In a paper published in 2017, Inge van Nistelrooij et al. [21] express the need to reframe autonomy in a shared decision-making process as relational autonomy. Authors state that, to reconceptualise relationality, it is mandatory to “turn to care ethics” [22].

Schuchter and Heller [23] also use notions of the ethics of care. They affirm that “the solution” to a moral problem does not lie in judging actions on the basis of moral principles, but in intensifying relationships and enhancing empathetic involvement*"*.

The need to manage moral issues, such as respect for a broader meaning of autonomy, the central role of the patient’s concept of dignity, the role of choice, the importance of truth, the concept of quality of life, the value of emotions and the existential issue, is an integral part of the palliative care approach.

In this sense, we believe that Ethics of Care takes into consideration aspects that classical ethics have overshadowed: trust and responsibility, protection of individuality, the context in which the relationship takes place, and the quality of the relationship.

This study is part of a larger project aimed at developing and implementing an ethics communication training programme addressed to HPs who treat patients with palliative care needs.

## Methods

The aim of this study was comprehending whether and how the ethics of care informs the way HPs make sense of and handle ethical issues in palliative care.

We employed a generic qualitative research design [24] using semi-structured interviews.

### Study population

We conducted the study in a clinical cancer centre in northern Italy. The study was approved by the Ethics Committee of the Provincial Health Authority of Reggio Emilia.

Eligible participants were physicians and nurses from eleven hospital wards who were involved daily in the care of patients with chronic diseases with poor prognoses and had previously attended a 4-h training on palliative care held by the hospital Palliative Care Unit. A conveniently selected sample of a physician and a nurse per ward was chosen.

The heads of each hospital ward were informed by the Principal Investigator (PI) on the objectives and the request for collaboration in the research. After obtaining access to the field, the PI e-mailed the information and request for participation to selected professionals. The invited participants were then contacted by telephone by the PI who, after obtaining consent, agreed on the place and times for participating in the study. In the cases of refusal to participate, the researchers contacted potential replacements. All participants provided signed informed consent to participate in the qualitative interviews.

Sixteen out of twenty subjects agreed to participate in the study. We interviewed 9 physicians and 7 nurses from 11 wards. The participant characteristics are shown in Table 1.

**Table 1:** Participant characteristics

Profession	Ward	Age range
Physician	Cardiology	51–60
Nurse	Cardiology	31–40
Physician	Rehabilitation	41–50
Nurse	Rehabilitation	41–50
Physician	Obstetrics and Gynaecology	61–70
Physician	Haematology	41–50
Nurse	Haematology	21–30
Physician	Internal medicine-Oncology	61–70
Physician	Oncology	31–40
Nurse	Oncology	31–40
Physician	Nephrology	41–50
Physician	Pneumology	31–40
Nurse	Pneumology	51–60
Nurse	Infection disease	41–50
Nurse	Intensive care	31–40
Physician	Long-term care	31–40

### Data collection

We derived the thematic areas to discuss during the interview sessions with participants from the ethics of care framework, consequentially focusing on care relationship.

Thematic areas were developed by the P.I. (LDP), a researcher and bioethicist, and SDL, a clinical psychologist expert in qualitative research. They agreed on three broad topics: the perception of ethical issues, the experienced role of ethical issues within the care relationship, the way interviewees recognise and deal with ethical dilemmas within the care relationships.

We used open-ended, semi-structured interviews [25] because of their flexible structure, which allows the interviewer to adapt and change the questions according to the interviewee’s agenda and answers. For conducting the interview, we pre-planned some exemplifying questions that we report in Table 2.

**Table 2:** Semi-structured interview guide

Thematic areas	Guiding questions
Perceiving ethical issues	When talking about the moral or ethical dimension of professionals regarding the relationship with patients suffering from an advanced-stage illness, what aspects do you think about? What comes to your mind?
	Thinking about your professional experience, what ethical principles do you mainly consider?
Comprehending the role of ethical issues within the care relationship	Which strategies do you usually use to explore the existential values and priorities of a patient?
	In your relationship of care with patients, how much consideration do you place on what the patient has expressed is important for him or her?
	Thinking about your experience in caring for patients suffering from an advanced-stage illness, what role does the patient’s moral conception have in the care relationship?
Recognising and dealing with ethical dilemmas within the care relationship	In your professional experience did you ever deal with situations in which you felt you had to question your moral principles?
	Can you provide an example of a problem that you would describe as “ethical” and that threw you into a moral crisis?
	If you are faced with a moral dilemma, which resources would you use to tackle it?

The P.I. conducted the semi-structured individual interviews. She did not know the participants.

The semi-structured individual interviews lasted a mean of 45 min.

### Data analysis

Interviews were audio-recorded and transcribed verbatim. Data analysis was conducted by the P.I, together with S.T., palliative care physician with experience in qualitative research, and L.G, qualitative research methodologist. We performed a theoretically driven thematic analysis [26] by following these analytical stages:

- L.D.P. transcribed the interviews verbatim and shared the transcripts with colleagues. They wrote comments and initial thoughts in a memo;
- L.D.P., S.T. and L.G. extracted portions of the text individually and then shared their work to reach an initial agreement. During this stage, they inductively conducted the thematic analysis [26], providing their insights;
- subsequently, they mapped the themes onto the ethics of care framework;
- they independently reviewed themes and allocated portions of the text to the newly reconfigured themes;
- together, they re-defined themes and re-named them to achieve internal consistency;
- L.D.P. selected representative extracts from the interviews and drafted the final report, which was checked and amended by all the authors.

## Results

Sixteen out of twenty subjects agreed to participate in the study. We interviewed 9 physicians and 7 nurses from 11 wards. They were 10 female and six male; the mean age was 43,8 years old (range 21–70).

Five themes and related sub-themes have been identified: 1) morality is providing general care; 2) morality is knowing how to have a relationship with patients; 3) morality is recognizing moral principles; 4) moral dimension and communication; and 5) moral dilemma as individual conflicts. Themes and sub-themes are shown in Table 3.

**Table 3:** Overview of themes and related sub-themes

Themes	Sub-themes
1) Morality is providing general care	Its crucial role in the care relationship
	Morality as respecting patient’s dignity and values
2) Morality is knowing how to have a relationship with patients...	… through emotionality
	… dealing with personal involvement
3) Morality is recognizing moral principles	Autonomy
	Humanity in care
4) Morality is giving importance to dialogue and communication	Ability to listen
	Ability to give meaning to the patient’s narrative
5) Moral dilemma as individual conflicts	Personal sphere

### Morality is providing general care

Morality plays a crucial role in the relationship of care, which cannot be demanded and cannot be avoided.

“Morality is the first hurdle we face, together with ethics and deontology. Deontologically, it is the sick person who is at the centre of the care, and morally, one should try to work in an ethical manner, understood as good behaviour…. but these concepts do not always go hand in hand” (P01).

Morality emerges as the human side of care and deals with giving importance to aspects such as knowing how to tell the truth, knowing how to answer questions on the sense and meaning of suffering, and being able to have a dialogue with the patient. Respect for patient dignity and his or her values is the manifestation of morality in the care relationship. Although it is expressed in different ways, due to the different roles they play, morality has the same meaning for nurses and physicians, making the care truly global.

“Morality is respect for everything, the care of the patient’s morality, the care of everything, […]. I think that all professionals should first of all respect themselves, and then give this respect to others” (N02).

“I believe that there are ways or strategies to talk about morality, but we don’t have them. This is what is missing. But you realise that it is often enough just to listen to, and when you give answers, to give these with your heart” (N06).

“If I think of morality, I think of my professional ethics, which is expressed in giving the best from a scientific point of view, and then entering into empathy with patients, so that they feel at ease in a complex path of care and, finally, in creating a relationship of trust” (P05).

### Morality is knowing how to have a relationship with patients

The relationship is an essential aspect of care, intended in a moral sense, and must involve all “actors” of the care process: patients, relatives and HPs. This perspective is very clear in some interviewees:

“I believe that everything revolves around a relationship based on affection. This type of affection must be transmitted in some way at every stage. And this is done through words, gestures, physical contact […]. You must know how to be in the relationship.” (P11)

“It is difficult to abstractly establish how to behave in certain situations with real protocols. However, in my opinion, some techniques, even relational ones, can certainly help. Although, we do not all agree on this point” (P14).

Knowing how to be in the relationship, knowing how to manage it, and considering it in emotional terms, emerges as a way to provide care. Some participants report that the relationship cannot become too personal, and a certain amount of professionalism must always be maintained. For this reason, the relationship is difficult, challenging and, as it is built, it must be nourished daily. Others conceive of personal involvement as a limit in the care relationship; although unavoidable, it comes with the risk of being overwhelmed.

“Involvement is always there. But it is not that kind of involvement that makes you say: “I will bring the pain of that patient home with me,” it consists of entering into a challenging and demanding relationship with that person” (N09).

“As soon as you establish a dialogue with the patient on moral issues and find out what is important to him/her, you enter into the patient’s subjective sphere which you must be able to perceive and manage” (P03).

### Morality is recognizing moral principles

HPs show that they have a broader idea of the moral principles featuring the care relationship compared to being strictly principlist. Nevertheless, sometimes the definition of these principles is not entirely clear. The principle of autonomy, for example, was directly mentioned only once, and yet in what the interviewees reported, the influence of this regulatory principle appears evident:

“My first principle is to make people aware, to try and give a person the tools so that they can make an autonomous and independent choice” (P07).

“The principles that guide me are those of respect, of the attempt to understand the patients’ experience and trying to understand and evaluate their situation” (N10).

“Morality is respect for the patients’ way of thinking, their decisions and values, the ability to not make them suffer, to eliminate everything that is harmful by meeting their needs, even if it goes against what I think” (N08).

Relational autonomy, correctness, sincerity and humanity are among the moral principles that are most often highlighted:

“I would say, first of all, that we are talking about the human side of care. Yes, I would say the human and relational component. And then the honest side of care. Morality concerns the humanity in a relationship of care” (P12).

### Morality is giving importance to dialogue and communication

Interviewees talk about morality through the different skills they use to put it into practice. These skills deal with the ability to dialogue and to listen to the patient, to give meaning to the patient’s narrative, to share his/her values, and to personalise communication exchanges; moreover, the professional awareness that telling the truth is not a univocal process, strongly emerges from the interviews.

“My strategy is to listen to, to explore the dimension of the sick patient’s existence, trying to understand how much that person is still anchored in his/her life […]. The patient’s value horizon guides the communication” (P15).

“Morality has many aspects, even of a personal and cultural nature. There is the way that you conceive your own morality and that of the patient. You have to learn to talk about it” (N13).

“To explore the values of a patient, it is important to understand their life experiences, their beliefs and interpretations” (P04).

“You also need to be able to see a desire, a wish emerging from the fragments of speech of the sick person. It is important for the communication to be gradual, to understand what truth is acceptable, and to know how to communicate it. The discourse of truth is a moral discourse, for example” (P16).

### Moral dilemmas as individual conflicts

All interviewees define moral dilemma as an inner conflict, to which they frequently cannot find a solution or that they cannot manage; therefore, it’s not unusual that dilemma often remain unresolved and accepted as an inevitable aspect of healthcare profession. Some participants refer to moral dilemmas highlighting their difficulty in reading end-of-life situations.

The narrated dilemma often touches on a very personal sphere: rather than concerning deontology or a specific ethic framework, it is embodied in the life experience of each individual professional.

“I prefer to help young people with cancer and their suffering as quickly as possible, perhaps by means of terminal sedation. On the other hand, my Christian ethics tell me: “What are you thinking? It is not up to you to decide it”. Therefore, many times my decision, though painful, is somewhere between a treatment that alleviates suffering and the respect of my Christian ethics” (P15).

“It concerned a personal situation, with my father […]. I lied to him about whether he was going to die. I felt very bad and after 25 years I still don’t know if it would have been better to tell him, he would have died anyway… If he had been one of my patients, I would have told him, but it’s different with family members…” (P12).

“I go into crisis when family members ask me not to tell the truth to the patients. I mean, if I was in their position, I would want to know, I would want to make the decisions together with the doctor. I would like to choose how to live my life to the end” (N08).

“I go into crisis when I have to say that there are no more useful tools to cure them, then I invent atypical drugs, nothing special, but in practice we continue to treat the patient to give the illusion that we are doing something” (P16).

## Discussion

The aim of this study was to comprehend whether and how the ethics of care informs the way HPs make sense of and handle ethical issues in Palliative care.

In our findings, morality fully emerges as a multidimensional concept. Its different meanings can be summarised by the following themes: morality is providing general care; it is knowing how to have a relationship with patients; it means recognizing moral principles and giving importance to dialogue and communication. Moreover, HPs seems to perceive moral dilemmas as “inner conflicts” that they cannot manage.

Although morality arise as an unconscious and unstructured concept, it seems to play a significant role in the care relationship. No explicit reference emerges in favour of a single ethical framework used in daily clinical practice; HPs talk about ethical issues in palliative care using notions and concepts such as caring relationship, listening, dialogue. These aspects are strongly highlighted in the ethics of care approach, focusing - as Leget wrote – on the caring relationship as being constituted of both patient and professional, as well as on the larger context of a person’s life [20].

Ethics emerges as an aspect of care concerning not only existential issues at the end of life, but also a number of choices throughout the entire patient care pathway. These choices have to deal with patient’s comfort, body care, patient’s preferences toward the administration of treatments.

From our results, it emerges that HPs tend to balance patient empowerment, compassion and understanding with solicitude within the care relationship. Thought compassion or solicitude are key concepts not only of the ethics of care approach, nevertheless they address specific caring attitudes described by the ethics of care approach, i.e., telling the truth while keeping hope alive, respecting as much as possible the degree of patient autonomy and meeting the patient’s spiritual needs, especially at the end of life [4, 27–29].

Our results seem to confirm the need for HPs for a step-by-step moral training. In fact, they tend to approach ethical issue with great emotional involvement, sometimes reporting on personal events; in addition, they seem to lack skills aimed at resolving dilemmas.

Without oversimplifying matters, principlism can help in reasoning about classical ethical principles and their application to a single moral dilemma [7]; the ethics of virtue can help in developing moral attitudes and “practical wisdom” [30]; the ethics of care underlines the importance of intensifying relationship and enhancing empathetic involvement [23]. These approaches, taking together, can be the basis for the development of a moral training providing HPs with ethical communication skills to interpret moral problems in a plural way.

As Leslie Bender [31] demonstrates, ethics is giving importance and focusing on care, compassion, availability, dialogue and communication, as well as learning the ability to listen carefully to others and to pay attention to the needs of others.

### Strengths and limitations

The research was consistently designed and conducted as a theory-driven study: the ethics of care theory formed the basis of all the steps (from the definition of the study design to the construction of the interview guide and data analysis), and this made a contribution to the transparency. We are fully aware that biases may arise from a prestructured qualitative research [35] design, but the choice of conducting this type of study depended on several methodological choices and organisational constraints: the scarceness of qualitative research in this context, the time and resources available, the purpose of proving the relevance of ethics of care in practice, and a data analysis process that is coherent with the purpose.

Among methodological limitations, we should highlight the following. Interviews were conducted by one interviewer only. However, data were analysed and discussed by a multidisciplinary team of researchers, and this could ensure scientific rigour and intersubjective corroboration. Since the study included only sixteen participants for convenience, we could not evaluate saturation. Nonetheless, we recruited both physicians and nurses from ten different hospital wards, allowing us to maximise and vary the professional perspectives included in the study.

## Conclusions

The results of this study suggest that for Health Professionals recognizing moral principles, dealing with ethical dilemmas and giving importance to dialogue and communication is paramount in the care relationship.

This requires developing and implementing effective educational programs focused on step-by-step moral training. The program should include at least the following objectives: empowering HPs with the ability to recognise ethical dilemmas and analyse conflicts; promoting sensitiveness to principles, values, goals and wishes of patients; and ensuring the ability of HPs to come to reasoned decisions in daily clinical practice [32–34].

Different ethical approaches can help in reaching the objectives described; the ethics of care framework also includes the belief systems of HPs; moreover, it allows the values of the patients and HPs to come to light through the relationship of care.

## Data Availability

The datasets used and analysed during the current study are available from the corresponding author on reasonable request.

## Abbreviation

HPs: Healthcare Professionals
